# Prof. Ives

**Published:** 1861-11

**Authors:** 


WE regret to announce the death of the venerable PROFESSOR IVES, of Yale College, well known for a long series of years as an able and successful practitioner and professor. His graduation at Yale was nearly a third of a century since, and he became a professor almost half a century ago. He died in New Haven, at the age of eighty-four.

